# The Prognostic Role of Klotho in Patients with Chronic Kidney Disease: A Systematic Review and Meta-analysis

**DOI:** 10.1155/2019/6468729

**Published:** 2019-06-02

**Authors:** Qi-feng Liu, Li-xia Yu, Jian-hua Feng, Qiang Sun, Sha-sha Li, Jian-Ming Ye

**Affiliations:** ^1^Department of Nephrology, Kunshan First People's Hospital Affiliated to Jiangsu University, 91 Qianjin West Road, Kunshan, Jiangsu 215300, China; ^2^Clinical Research & Lab Centre, Kunshan First People's Hospital Affiliated to Jiangsu University, 91 Qianjin West Road, Kunshan, Jiangsu 215300, China

## Abstract

**Objective:**

The prognostic role of Klotho in patients with chronic kidney disease is still controversial. Therefore, we performed this meta-analysis to assess the relationship between the low sKlotho level and the risk of adverse kidney outcomes.

**Materials and Methods:**

We systematically searched medical databases, such as PubMed, Embase, and the Cochrane Library, for eligible publications regarding the relationship between the low sKlotho level and risk of adverse kidney outcomes. The quality of included studies was assessed by using the Newcastle–Ottawa Scale. Combined hazard ratios (HRs) and its 95% confidence intervals (CIs) were calculated using a random- or fixed-effect model. Subgroup analysis was conducted with stratification by age, estimated glomerular filtration rate (eGFR), follow-up interval, region, and study quality. All data was analyzed by RevMan 5.3 analysis software.

**Results:**

Eight cohort studies with 3586 participants from 3818 studies were included in our final analysis. Levels of sKlotho were significantly correlated with the eGFR, with a summary correlation coefficient *r* and 95% CI of 0.469 (0.226, 0.658). Additionally, low sKlotho levels were strongly associated with increased adverse kidney outcomes, and the pooled HR and its 95% CI were 1.64 (1.19, 2.26; *P* = 0.002), despite publication bias and statistical heterogeneity (*I* ^2^ = 52%, *P* = 0.07). Furthermore, this positive correlation was still observed in all of the subgroup analyses. However, heterogeneity was present in subgroup analyses stratified by the eGFR and follow-up interval.

**Conclusion:**

Levels of sKlotho are positively correlated with the eGFR, and low sKlotho levels are significantly associated with an increased risk of poor kidney outcomes. Therefore, sKlotho could be used as a novel biomarker for early diagnosis and prognostic assessment for patients with chronic kidney disease. Studies with a larger sample size and longer follow-up period are warranted to validate our results.

## 1. Introduction

Kuro-o et al. identified *α*-Klotho in 1997 as a novel antiaging gene [1], encoding two Klotho proteins. One of these proteins is the membrane-bond form (mKlotho), a single-pass membrane protein, which is expressed on the cell surface. The short extracellular domain of mKlotho can be cleaved by proteases and released into the blood. This shed extracellular domain is defined as soluble or secreted Klotho (sKlotho) [2]. mKlotho and sKlotho possess distinct biological functions [3]. mKlotho forms a complex with the fibroblast growth factor receptor (FGFR) and serves as the coreceptor for the fibroblast growth factor 23 (FGF23) to maintain mineral homeostasis [3, 4]. sKlotho can be detected in the circulation by ELISA assay [5] and is believed to be a main active form. sKlotho exerts pleiotropic beneficial effects by acting as a circulating hormone and protects cells against oxidative stress, hypoxia, and inflammation and inhibits cell apoptosis and organ fibrosis [6]. The Klotho gene is located in multiple organs including the kidney, brain, parathyroid, testis, and pituitary gland [1, 7, 8]. Among these organs, the kidney has the highest Klotho levels, indicating that the kidney is the major organ which generates Klotho [9]. Therefore, unsurprisingly, Klotho levels decrease if the organ of origin is diseased [10, 11].

Chronic kidney disease (CKD) is increasingly considered as a major public health issue worldwide with high mortality and morbidity rates [12]. Currently, there is no effective therapy available for treating CKD. Therefore, early detection or prognosis is important for the prevention and treatment of CKD. However, there is no standard biomarker for early diagnosis and the monitoring of disease exacerbation in the course of CKD. Emerging evidence from patients with CKD has shown that sKlotho levels are decreased in the early stages of CKD, and they further decline as CKD progresses [11, 13–15]. Moreover, reduced sKlotho levels are associated with an elevated risk of deterioration in renal function or renal replacement treatment (RRT) [13, 16]. Therefore, sKlotho is proposed as a biomarker for the early diagnosis and progression of CKD. A correlation between sKlotho levels and kidney function has been recently systematically reviewed by Wang et al. [17]. These authors showed a positive association between sKlotho levels and the estimated glomerular filtration rate (eGFR), and they evaluated the possibility of sKlotho as an early biomarker for CKD early diagnosis. However, the role of sKlotho in predicting adverse outcomes in the kidney remains controversial [18]. Therefore, we performed a meta-analysis to assess the prognostic role of sKlotho by investigating the association between sKlotho levels and progression of CKD.

## 2. Materials and Methods

### 2.1. Search Strategy

A systematic literature search of PubMed, Embase, and the Cochrane Library was performed by two authors. The search was restricted to articles written in English. The terms that were used for the search were as follows: ([Klotho or alpha-Klotho or *α*-Klotho or *α*KL] and [chronic kidney disease or CKD or chronic kidney insufficiency or chronic kidney failure or chronic nephropathy or chronic kidney dysfunction] or [biomarker or marker or prognosis or outcome or progression or decline or deterioration]). Moreover, the reference lists of included studies were also retrieved manually for additional relevant studies. The updated date was January 15, 2019.

### 2.2. Inclusion Criteria and Exclusion Criteria

We included studies on the basis of the inclusion and exclusion criteria. Inclusion criteria were as follows: (1) a cohort or cohort and observational study, (2) a study that investigated the association between sKlotho levels and adverse kidney outcomes in patients with CKD, and (3) the patient's age was ≥18 years. Exclusion criteria were as follows: (1) an observational study, (2) a study that investigated the relationship between renal Klotho or urinary sKlotho levels and kidney function or other parameters, (3) a study with incomplete data, (4) patients with kidney transplantation or dialysis, (5) an animal experimental study (*in vivo* or *in vitro*), and (6) case reports, posters, editorials, and reviews.

### 2.3. Study Selection

Two authors independently screened the abstracts and titles of the relevant studies and eliminated studies that were not applicable according to the prestated inclusion criteria. However, reviews that possibly contained relevant information were initially included. The same two authors independently assessed the eligibility of the remaining full-text articles. Disagreements regarding study selection were resolved by discussion with an arbitrator.

### 2.4. Data Extraction

Two investigators extracted data independently from the included literature using a standardized data extraction form. The extracted content for each study included the first author's name, year of publication, study design, sample size, age, research region, assay use, correlation coefficient (Pearson or Spearman), hazard ratio (HR), odds ratio (OR), and 95% confidence interval (CI). The estimated HRs were acquired from the Kaplan–Meier curves as previously described [19] if the HRs were not obtained directly in the studies. Discrepancies in data extraction were addressed by consulting a third arbitrator. The most complete data were used if more than one publication of one study existed. If the data were not obtained or not complete, the first or corresponding author was contacted by e-mail.

### 2.5. Quality Assessment

The quality of included studies was independently assessed by three authors using the Newcastle–Ottawa Scale (NOS) [20]. Studies with ≥8 awarded stars were considered as high-quality studies. Disagreements among authors were resolved by discussing with an independent third party. The quality items assessed were eight items including patient selection, comparability, and outcome.

### 2.6. Meta-analysis

Pearson correlation coefficients were converted into Spearman correlation coefficients, and the latter were used for estimating the associations between sKlotho levels and the eGFR [17, 19]. Correlation coefficients underwent Fisher's *Z* transformation to generate a *Z* value, and then we calculated the standard error of *Z*. Meta-analysis was used to obtain the summary Fisher's *Z* value, and this was then transformed back by inverse Fisher's transformation to obtain the summary effect size (*r*) and 95% CIs. The pooled HRs and corresponding 95% CIs were used to evaluate the effect of sKlotho levels on adverse kidney outcomes. Meta-analysis was performed by Review Manager 5.3 analysis software (Cochrane Collaboration, Copenhagen, Denmark). Heterogeneity across included studies was analyzed using *I* ^2^ statistics. The fixed-effect model was used when the *I* ^2^ value was <50%. The random-effect model was applied when the *I* ^2^ value was >50%. Subgroup analysis was performed to examine the source of heterogeneity. The stability of the results was evaluated by sensitivity analysis via switching between the fixed-effect and the random-effect model. Potential publication bias was assessed by using the funnel plot.

## 3. Results

### 3.1. Study Selection

**Figure 1:**
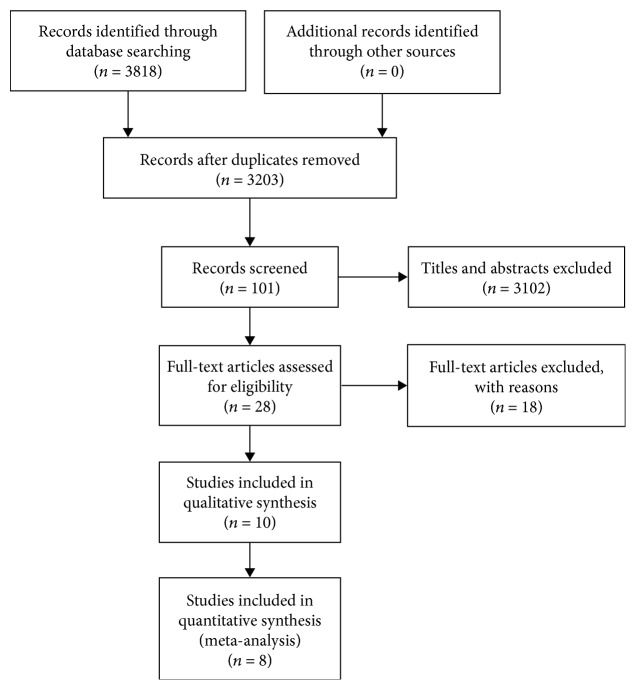
Flow chart of the included studies in the meta-analysis.

A total of 3818 relevant publications were extracted by searching databases, including PubMed, Embase, and the Cochrane Library. Of these studies, 615 were removed because of duplication. A total of 3102 publications were excluded by screening titles and abstracts. Ten studies were included by reviewing the full text, and eight studies were finally identified for our meta-analysis on the basis of the inclusion and exclusion criteria. The article selection process is shown in Figure 1.

### 3.2. Characteristics of Included Studies

Eight cohort studies were included in our final analysis of 3586 participants [13, 16, 18, 21–25]. Three studies provided information on the sKlotho level and the eGFR (Pearson correlation coefficients or Spearman correlation coefficients) [13, 16, 25], and two studies reported data on sKlotho and an annual decline in eGFR (Pearson correlation coefficients) [21, 24]. Four studies reported data on the sKlotho level and kidney outcomes (HR and its 95% CI) [13, 18, 24, 25]. HR was extrapolated from the Kaplan–Meier curves in one study [23]. Moreover, OR was estimated on the basis of data that were provided in another study [22] because its HR could not be obtained directly or calculated indirectly. Three studies reported correlation coefficients for FGF23 and eGFR [13, 21, 25]. Two studies reported HRs and 95% CIs for FGF23 levels and kidney outcomes [18, 25]. Characteristics of included studies are displayed in Table 1. Quality of the included studies was assessed by the Newcastle–Ottawa Scale for cohort studies. Specific scores that ranged from 4 to 9 are shown in Table 2, and the average score was 7.1.

### 3.3. Association of sKlotho Levels and the eGFR

**Figure 2:**
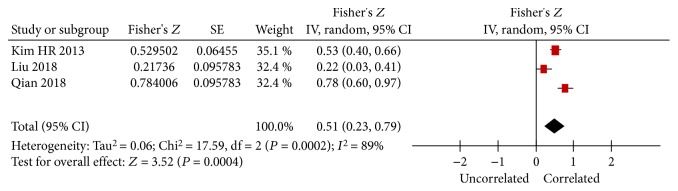
Forest plots of the summary Fisher's *Z* value with its 95% CIs for the association between sKlotho and eGFR. Summary correlation coefficient (*r*) and 95% CI were 0.469 (0.226, 0.658) by inverse Fisher's transformation.

One study showed no relationship between sKlotho and the eGFR (the correlation coefficient was not shown in this study) [18], and three studies reported data on the association between sKlotho and the eGFR in the cross-sectional analysis [13, 16, 25]. The remaining four studies did not report any data on sKlotho and the eGFR. All of the three studies demonstrated a positive correlation between sKlotho levels and the eGFR. Meta-analysis showed that the combined Fisher's *Z* value with the corresponding 95% CI was 0.51 (0.23, 0.79; *P* < 0.001; Figure 2). After inverse Fisher's *Z* transformation, the summary *r* and its 95% CI were 0.469 (0.226, 0.658). Our findings suggested that sKlotho levels were positively associated with the eGFR. The random-effect model was used because of significant heterogeneity (*I* ^2^ = 89%, *P* = 0.0002, Figure 2). Funnel plots for these studies showed a symmetrical distribution and indicated that there was no publication bias (data not shown). To investigate the source of heterogeneity, we recalculated the combined results by excluding one study each time, and statistical heterogeneity still existed.

**Figure 3:**
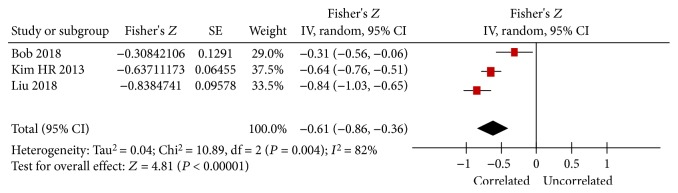
Forest plots of the summary Fisher's *Z* value with its 95% CIs for the association between FGF23 and eGFR. Summary *r* and 95% CI were -0.544 (-0.696, -0.345) by inverse Fisher's transformation.

Three studies reported data on FGF23 levels and the eGFR, and all of the three studies showed a negative association between FGF23 levels and the eGFR [13, 21, 25]. Our combined Fisher's *Z* value with its 95% CI was −0.61 (−0.86, −0.36), despite significant heterogeneity (*I* ^2^ = 82%, *P* = 0.004, Figure 3), and the calculated summary *r* and its 95% CI were −0.544 (−0.696, −0.345).

### 3.4. Association of sKlotho Levels and an Annual Decline in the eGFR

Only two studies reported data on sKlotho levels and an annual decline in the eGFR [21, 24]. In Kim et al.'s study, sKlotho levels were negatively correlated with an annual decline in the eGFR (*r* = −0.217, *P* = 0.009) after adjustment for clinical parameters including baseline eGFR [24]. However, in Bob et al.'s study, sKlotho was positively associated with an annual decline in the eGFR (*r* = 0.714, *P* = 0.0004) and one increase with a standard deviation in the sKlotho level followed by an augmentation with 0.623 standard deviation in the decline of eGFR adjusting for confounding factors [21]. However, high heterogeneity was observed in these two studies (*I* ^2^ = 98%, *P* < 0.001) when the data were combined. Due to high heterogeneity and the small number of studies, meta-analysis was not performed further to clear up this confusion.

### 3.5. Association of sKlotho Levels and Adverse Kidney Outcomes

**Figure 4:**
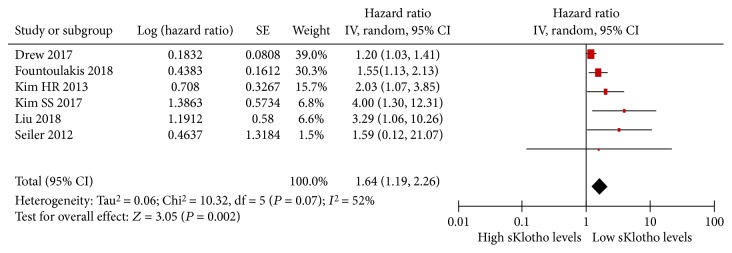
Forest plots of low sKlotho levels and adverse kidney outcomes from the included six studies.

Of the included eight studies, one study reported that a change in sKlotho levels (ΔsKlotho level) was an independent predictor for an increased risk of RRT after adjusting for confounders [16]. However, one study showed that a high sKlotho level was significantly associated with a rapid decline in the annual eGFR after adjusting for confounding factors [21]. Because HRs or ORs could not be obtained or calculated in these two studies, they were excluded in our meta-analysis. Six studies with 3419 participants were identified as being eligible for our meta-analysis [13, 18, 22–25]. Their HRs or ORs were obtained directly or indirectly after adjustment for possible confounding factors including basal eGFRs. In the six studies, one study showed no relationship between reduced sKlotho levels and increased adverse kidney outcomes (doubling of serum creatinine (Scr) levels or RRT) [18] and the remaining five studies showed a strong correlation [13, 22–25]. With the random-effect model, the pooled HR and its 95% CI were 1.64 (1.19, 2.26; *P* = 0.002; Figure 4), which suggested that low sKlotho levels were significantly associated with increased adverse kidney outcomes. No studies were found to have a significant effect on the total results of this meta-analysis. The distribution of funnel plots was not symmetrical, which suggested that there was publication bias.

**Figure 5:**
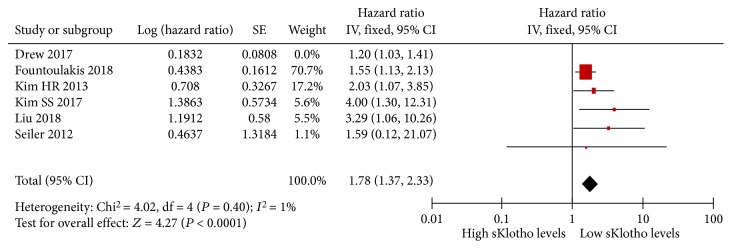
Forest plots of low sKlotho levels and adverse kidney outcomes after removing Drew et al.'s study (2017).

There was moderate heterogeneity in the result of the meta-analysis of the included 6 studies (*I* ^2^ = 52%, *P* = 0.07, Figure 4). Sensitivity analysis showed that there was no heterogeneity (*I* ^2^ = 1, *P* = 0.40, Figure 5), and the total combined results were not altered after excluding Drew et al.'s study [22] (pooled HR, 1.78 (1.37, 2.33)). Therefore, Drew et al.'s study was the source of statistical heterogeneity. However, we eventually included this study because of its longest follow-up interval and largest sample size. To further search for the potential causes of significant heterogeneity across the studies, we conducted subgroup meta-analysis on the basis of average age (≥65 years or <65 years), eGFR (≥60 ml/min or <60 ml/min), follow-up interval (≥2 years or <2 years), research region (Asia or other countries), and study quality (score ≥8 or <8). The results of the subgroup meta-analysis are shown in Table 3. Positive associations were still apparent and were significant in all subgroups. There was high heterogeneity regarding the eGFR (*I* ^2^ = 77%, *P* = 0.04) and follow-up interval (*I* ^2^ = 62%, *P* = 0.05). There was no statistical heterogeneity regarding the average age (*I* ^2^ = 25%, *P* = 0.26), research region (*I* ^2^ = 2%, *P* = 0.36), and study quality (*I* ^2^ = 48%, *P* = 0.15). Therefore, the eGFR and follow-up interval were thought to be the sources of heterogeneity among the studies.

**Figure 6:**
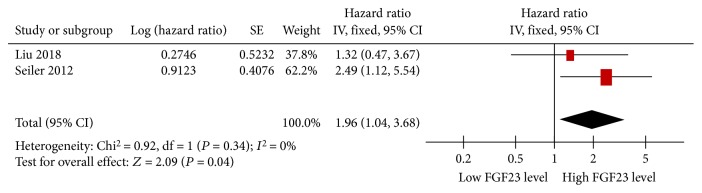
Forest plots of high FGF23 levels and adverse kidney outcomes.

Three studies showed data on FGF23 levels and adverse kidney outcomes [13, 18, 25]. High FGF23 levels predicted adverse kidney outcomes in two studies [13, 18], but a similar correlation was not observed in our recent study [25]. Because HR or OR was not obtained in Kim et al.'s study [13], this study was removed from our meta-analysis. The overall combined results showed that there was no heterogeneity between the two studies (*I* ^2^ = 0%, *P* = 0.34, Figure 6). Thus, meta-analysis was conducted further, and the pooled HR and its 95% CI were 1.96 (1.04, 3.68), indicating that high FGF23 levels were significantly associated with increased adverse kidney outcomes. Although there were a small number of enrolled studies, we believed that the result of the meta-analysis in homogeneous studies was stronger than that of the single original study.

## 4. Discussion

CKD and its complications are public health issues in the general population. Much effort has been made to screen and identify novel biomarkers for early diagnosis and prognostic estimation in patients with CKD. However, an ideal biomarker is still lacking. The novel antiaging factor sKlotho is a potential biomarker for CKD and has elicited considerable attention in recent years. Levels of sKlotho are primarily generated from the kidney, indicating that there is strong correlation between sKlotho levels and kidney function. Indeed, a number of human studies have suggested that sKlotho levels are not only associated with the state of kidney function but also reflect the extent of kidney injury. Shimamura et al. first reported that sKlotho levels began to decline from CKD stage 2 and continued to decline as CKD progressed [15]. Pavik et al. showed a 1 ml/min decrease in the eGFR accompanied by a 3.2 pg/ml decrease in sKlotho levels [14]. Our recent data also showed that sKlotho levels in CKD were decreased by 75% compared with those in healthy controls [25]. Similar findings have also been found in other human studies with CKD [26–28]. However, several studies have shown conflicting results. In these studies, sKlotho levels were not decreased but increased, or sKlotho levels across CKD stages were not significantly different in patients with CKD [18, 29, 30]. A recent meta-analysis by Wang et al. addressed this discrepancy [17]. In their study, the combined correlation coefficient *r* (between sKlotho and the eGFR) was 0.35 (0.23, 0.46, *P* < 0.05) [17], which suggested there is a positive correlation between sKlotho and the eGFR [17]. In their meta-analysis, eight studies were included with 1136 participants, and there was moderate heterogeneity (*I* ^2^ = 68.7%, *P* = 0.002) and no publication bias [17]. In line with this previous study, the pooled correlation coefficient *r* in our study was 0.469 (95% CI: 0.226, 0.658), which indicated that sKlotho levels are linearly related to the eGFR. However, our analysis only included three studies with a small sample size, there was significant heterogeneity, and the strength of our conclusion inevitably was limited. Therefore, our results should be interpreted with caution.

An increasing number of cohort studies have shown that progression of CKD or aggressive loss of the eGFR is significantly associated with sKlotho deficiency. Kim et al. observed that a 10 pg/ml increase was associated with a reduction by 4% in the risk of composite kidney outcomes, including RRT or doubling in Scr levels in patients with CKD stages 1–5 [13]. These authors showed that sKlotho levels below the median value had an increased risk of reaching combined endpoints (HR: 2.03; 95% CI: 1.07, 3.85) [13]. In patients with rapid loss of kidney function, we recently observed that low sKlotho levels were associated with an increased risk of doubling of Scr levels or RRT during follow-up (HR: 3.291; 95% CI: 1.056, 9.823) [25]. In patients with stable kidney function, Drew et al. showed that low sKlotho levels were persistently correlated with adverse kidney outcomes [22]. In this previous study, 2496 participants with a mean eGFR (74 ± 18 ml/min) were enrolled and were followed up for 3 or 10 years. Doubling of sKlotho levels was associated with reduced odds of decline in kidney function for a 30% decline in the eGFR (OR: 0.78; 95% CI: 0.66, 0.93) and for a 3 ml/min per year decline in the eGFR (OR: 0.73; 95% CI: 0.66, 0.99). Overall, low sKlotho levels (below the median value) were associated with a higher risk of reaching combined adverse kidney outcomes (calculated OR: 1.20; 95% CI: 1.03, 1.41) after adjusting for confounders. Because eGFR values were measured at one or two time points at the end of follow-up (3 or 10 years), thus, HR was not reported or calculated in this study. However, some studies provided inconsistent results that sKlotho levels failed to predict progression of CKD [18] and that high sKlotho levels were associated with a rapid decline in kidney function in patients with CKD [21]. Therefore, sKlotho's prognostic significance is still under extensive investigation. In the current study, we found that low sKlotho levels were associated with increased adverse kidney outcomes, which indicated that sKlotho could be a prognostic biomarker for patients with CKD. Notably, we found significant heterogeneity (*I* ^2^ = 52%, *P* = 0.07) in our study. However, significant heterogeneity was no longer present (*I* ^2^ = 1%, *P* = 0.4), and the pooled HRs were not altered after the exclusion of Drew et al.'s study [22]. This suggested stability of our meta-analysis results. However, association of sKlotho levels and an annual decline in the eGFR still remains uncertain due to the significant heterogeneity and the small number of studies. Therefore, further studies are needed to resolve the contradictory results.

The mechanism underlying low sKlotho levels increasing the risk of reaching adverse kidney outcomes is multifactorial. Oxidative stress, inflammation, and the renin–angiotensin–aldosterone system are risk factors that promote progression of CKD. Reduced sKlotho levels are also associated with enhanced oxidative stress and inflammation [31, 32]. Interestingly, upregulated Klotho levels facilitate the removal of reactive oxygen species by activating FOXO-mediated manganese superoxide dismutase [33]. Furthermore, upregulated Klotho levels inhibit inflammation by suppressing nuclear factor-*κ*B-mediated inflammatory processes in *in vivo* and *in vitro* studies [34, 35]. Additionally, sKlotho supplementation reduces renal angiotensinogen and angiotensin II levels, followed by the amelioration of renal fibrosis in diabetic and adriamycin nephropathy [36, 37]. Moreover, sKlotho therapy suppresses renal fibrosis by targeting several fibrotic signaling pathways, including TGF*β*-1/Smads and WNT/*β*-catenin signaling [37–39]. In our previous studies, sKlotho treatment inhibited renal fibrosis via suppression of endoplasmic reticulum stress or epithelial-mesenchymal transition [40, 41]. Because of the pleiotropic beneficial activities of sKlotho, it is a novel kidney-protective factor and treatment target for renal fibrosis [42]. Deficiency of sKlotho makes the kidney vulnerable to attacks from oxidative stress, ischemia, and inflammation, and this in turn aggravates kidney function. Therefore, loss of sKlotho is implicated in the development and progression of CKD, which is supported by our results.

FGF23 is primarily secreted by osteocytes and osteoblasts and was newly identified as a regulator of phosphorylation by forming a complex with Klotho [43]. FGF23 levels are increased in the early stages of CKD, and this even precedes the elevation of the parathyroid hormone and phosphate levels [43, 44]. As a compensatory response, elevated FGF23 levels are mostly ascribed to an increase in phosphate burden, and this maintains normal phosphorus levels in early CKD [44, 45]. As the eGFR continues to decline in the course of CKD, this compensatory mechanism fails to maintain phosphorus homeostasis, and this in turn leads to higher FGF23 levels [46]. Therefore, FGF23 is elevated in patients in the early stage of CKD, and this trend increases as CKD progresses because of persistent retention of serum phosphorus levels [47, 48]. FGF23 levels are inversely correlated with the eGFR, and they are emerging as an early biomarker for CKD in recent years [49–51]. Our findings are consistent with these previous findings that FGF23 is negatively correlated with the eGFR. Accumulating evidence has shown that increased FGF23 levels are strongly associated with an increased risk of adverse kidney outcomes or mortality in prospective studies on patients with CKD [13, 18, 52, 53]. This finding indicates that FGF23 may also have a prognostic value in these patients. However, our recent study showed conflicting results [25]. To address this issue, we also assessed the prognostic value of FGF23 in our included studies in the meta-analysis. We found that the pooled HR was 1.96 (95% CI: 1.04, 3.68) from two studies with 412 participants and there was no heterogeneity (*I* ^2^ = 0%, *P* = 0.34). Another study reported that a 10 pg/ml increase in FGF23 levels was related to a 4% increase of risk of adverse kidney outcomes and supported the strong correlation between the high FGF23 levels and increased risk of adverse kidney outcomes [13]. Taken together, our results showed that high FGF23 levels predicted adverse kidney outcomes in CKD patients with sKlotho loss. Therefore, an increase in FGF23 levels with a decrease in sKlotho levels may be associated with the development and progression of CKD [49]. However, the small number of enrolled studies inevitably reduced the power of our conclusion, and definite data are still lacking.

Above all, our meta-analysis of published longitudinal studies suggested that sKlotho levels were positively correlated with the eGFR and that a low sKlotho level predicted poor kidney outcomes in patients with CKD in adjusted analyses. These findings provide evidence for sKlotho as a potential biomarker for early detection and prognostic evaluation of CKD.

Our study has several limitations. First, there was significant heterogeneity. Sensitivity analysis showed that exclusion of Drew et al.'s study completely eliminated heterogeneity [22]. However, we eventually included this study because it represented the largest sample size and longest follow-up period among the included studies. We believe that the eGFR and follow-up interval, at least partly, are the sources of observed heterogeneity according to the results of subgroup meta-analysis. sKlotho is mainly produced by the kidney, and the state of kidney function affects sKlotho levels. Patients with an eGFR < 60 ml/min are always accompanied by lower sKlotho levels, and this condition is prone to suffering from more adverse outcomes. With regard to the follow-up interval, CKD progresses slowly under standard medical care and the risk of reaching outcomes is associated with the duration of follow-up. This means that the occurrence of adverse outcomes may depend on follow-up interval to some extent. Heterogeneity may also be caused by differences in patients' characteristics, including research region, sex, race, assay use, and other confounders. Moreover, the studies included are relatively few, especially the number of studies for FGF23 and renal outcomes, and the sample size is relatively small. Metaregression is not conducted further to investigate the source of heterogeneity. Therefore, these may have led to underestimation of our combined results, although the random-effect model was used for our analysis. Second, there was significant publication bias. Our meta-analysis included studies that were restricted to English publications. There may have been a few studies with negative results, a small sample size, or written in other languages that were not published. This inevitably resulted in publications bias. Third, some HRs in two studies were not reported in two studies [22, 23]. Instead, we had to extrapolate HR from the Kaplan–Meier curves [23] or calculate ORs according to the original data [22], and estimated HR or OR is less reliable. Fourth, the reference value for sKlotho has not been determined, and sKlotho levels are ranging from 326.4 to 2.44 ng/ml. The cut-off values of sKlotho are variable, and the method used for the Klotho assay varied in this meta-analysis. Moreover, the sKlotho level may be regulated by some drugs or the acute inflammatory process, which were not entirely excluded in the included studies. The fluctuations may have lessened the power of our results to some extent.

In conclusion, our meta-analysis shows that sKlotho levels are positively correlated with the eGFR. Moreover, low sKlotho levels are associated with an increased risk of reaching adverse kidney outcomes in patients with CKD. Our findings support the assumption that sKlotho could be used as a novel indicator for early diagnosis and prognostic assessment of CKD, despite the limitations discussed above. Prospective studies with larger sample sizes are still required to confirm our conclusions.

## Figures and Tables

**Table 1:** Characteristics of the included studies.

First author	Year	Country	Study design	Number	Follow-up period	Average age	Average eGFR(ml/min)	Low versus high sKlotho level	Outcomes	HR and 95% CI
Liu (Ref [25])	2018	China	Cross-sectional Prospective	112	20.1 ± 10.1 months	50.1 ± 14.0	38.2 ± 7.3 22.1 ± 6.3 10.8 ± 2.2	Median sKlotho level	Scr doubling RRT Death	Direct
Bob (Ref [21])	2018	Romania	Cross-sectional Retrospective	63	12 months	58.13 ± 12	65.2 ± 32.5	Overall sKlotho	Δdecline of eGFR	—
Fountoulakis (Ref [23])	2018	UK	Cross-sectional Prospective	101	9 (2-13) years	60 (40-82)	90.7 ± 20.0	Median sKlotho level	50% decline of eGFR Death	Indirect (estimated HR)
Qian (Ref [16])	2018	China	Cross-sectional Prospective	112	1.5 years	64.5 ± 12.7	—	ΔsKlotho level	RRT Cardio-cerebrovascular events	—
Drew (Ref [22])	2017	America	Prospective	2496	3 or 10 years	75 ± 3	73 ± 18	sKlotho quartile level	eGFR decline ≥ 30% or >3 ml/min per year	Indirect (estimated OR)
Kim (Ref [24])	2017	Korea	Prospective	147	32 (12-52) months	56.4 ± 10.8	93.0 ± 23.2	sKlotho tertile level	Annual eGFR decline Albuminuria	Direct
Kim (Ref [13])	2013	Korea	Cross-sectional Prospective	243	29.7 (6.0-62.1) months	45.7 ± 15.7	55.4 ± 36.5	Median sKlotho level	Scr doubling RRT Death	Direct
Seiler (Ref [18])	2013	Germany	Cross-sectional Prospective	312	2.2 ± 0.8 years	65.5 ± 12.1	43.8 ± 15.6	sKlotho tertile level	RRT Death	Direct

Abbreviation: RRT: renal replacement therapy; Scr: serum creatinine; HRs: hazard ratios; CI: confidence interval; eGFR: estimated glomerular filtration rate; Ref: reference.

**Table 2:** NOS scores of the cohort studies included.

Cohort study	Selection representativeness of the exposed cohort	Selection of the unexposed cohort	Ascertainment of exposure	Outcome of interest not present at start of study	Comparability control for important factor or additional factor^∗^	Outcome assessment	Was follow-up long enough for outcomes to occur	Adequacy of follow-up of cohorts	Total quality scores
Liu 2018	/	★	★	★	★	★	/	★	6
Bob 2018	/	★	★	★	/	★	/	/	4
Fountoulakis 2018	/	★	★	★	★★	★	★	★	8
Qian 2018	/	★	★	★	★★	★	/	★	7
Drew 2017	★	★	★	★	★★	★	★	★	9
Kim 2016	/	★	★	★	★	★	★	★	7
Kim 2013	/	★	★	★	★★	★	★	★	8
Seiler 2012	/	★	★	★	★★	★	★	★	8

Note: ^∗^2 stars could be awarded for this item. Studies that controlled for age or eGFR were awarded one star, respectively. Abbreviation: NOS: Newcastle–Ottawa Scale.

**Table 3:** Results of subgroup analysis about the association between Klotho and renal outcomes.

Subgroup	Studies	Statistical method	Heterogeneity	Effect estimate	*P* value
Age	6	HR (IV, fixed, 95% CI)		1.33 (1.16, 1.53)	<0.01
Age ≥ 65	2 (Ref [18, 22])	HR (IV, fixed, 95% CI)	*P* = 0.83; *I* ^2^ = 0%	1.20 (1.03, 1.41)	0.02
Age < 65	4 (Ref [13, 23–25])	HR (IV, fixed, 95% CI)	*P* = 0.26; *I* ^2^ = 25%	1.79 (1.37, 2.33)	<0.01
eGFR	6	HR (IV, random, 95% CI)		1.81 (1.28, 2.55)	<0.01
eGFR ≥ 60 ml/min	2 (Ref [22, 24])	HR (IV, random, 95% CI)	*P* = 0.04; *I* ^2^ = 77%	1.92 (0.61, 6.05)	0.04
eGFR < 60 ml/min	4 (Ref [13, 18, 23, 25])	HR (IV, random, 95% CI)	*P* = 0.40; *I* ^2^ = 1%	1.79 (1.37, 2.34)	<0.01
Follow-up interval		HR (IV, random, 95% CI)		1.64 (1.19, 2.26)	<0.01
Follow − up ≥ 2 years	4 (Ref [13, 22–24])	HR (IV, random, 95% CI)	*P* = 0.05; *I* ^2^ = 62%	1.56 (1.12, 2.17)	<0.01
Follow − up < 2 years	2 (Ref [18, 25])	HR (IV, random, 95% CI)	*P* = 0.61; *I* ^2^ = 0%	2.92 (1.03, 8.28)	0.04
Research region	6	HR (IV, random, 95% CI)		1.64 (1.19, 2.26)	<0.01
Asia	3 (Ref [13, 24, 25])	HR (IV, random, 95% CI)	*P* = 0.52; *I* ^2^ = 0%	2.55 (1.55, 4.20)	<0.01
Other countries	3 (Ref [18, 22, 23])	HR (IV, random, 95% CI)	*P* = 0.36; *I* ^2^ = 2%	1.27 (1.10, 1.47)	<0.01
Study quality	6	HR (IV, random, 95% CI)		1.64 (1.19, 2.26)	<0.01
High-quality study	4 (Ref [13, 18, 22, 23])	HR (IV, random, 95% CI)	*P* = 0.26; *I* ^2^ = 26%	1.37 (1.11, 1.69)	<0.01
Low-quality study	2 (Ref [24, 25])	HR (IV, random, 95% CI)	*P* = 0.81; *I* ^2^ = 0%	3.63 (1.63, 8.08)	<0.01
